# RETRAUMATIZATION WHEN AN ADULT CHILD CARES FOR THEIR PARENTAL PERPETRATOR THROUGH SERIOUS ILLNESS OR THE END OF LIFE

**DOI:** 10.1093/geroni/igad104.0958

**Published:** 2023-12-21

**Authors:** Jaime Goldberg, Jooyoung Kong

**Affiliations:** University of Wisconsin-Madison , Madison, Wisconsin, United States; University of Wisconsin-Madison, Madison, Wisconsin, United States

## Abstract

Current trauma-related literature does not readily include the experiences of the caregiving population. While the caregiving literature does recognize that caregivers experience trauma, it has not yet explored when the caregiver’s source of trauma is the care recipient. To begin to address this gap, this research explores the retraumatization adult survivors of parental childhood maltreatment may experience when they become caregivers for their parental perpetrator during serious illness or the end of life. Participants were recruited through purposeful sampling techniques, via social media and word-of-mouth. Using a phenomenological approach, 22 semi-structured, virtual interviews were conducted; 13 participants identified retraumatization as part of their caregiving experience. Focused content analysis revealed three subthemes under the umbrella of retraumatization: 1) reminders of past abuse; 2) re-experiencing abusive behaviors; and 3) physical contact with the body of the former abuser. Results suggest that memories of past abuse, ongoing abusive behavior, and proximity to the parental perpetrator’s body evoke feelings of fear/anxiety, worthlessness, shame, anger, and disgust in participants, reminiscent of the feelings they experienced in childhood. Taking on the roles/responsibilities of caregiving triggered these feelings unexpectedly, as the adult children were now interacting with their parent more frequently and in new/more intimate ways. These findings invite health and mental health professionals to have heightened awareness of and sensitivity to this often-overlooked population and use trauma-informed assessment and intervention techniques to support caregivers’ health and well-being. Results can inform future research at the nexus of caregiving and trauma.

